# High Frequency Dielectric Characteristics of Electrochromic, WO_3_ and NiO Films with LiNbO_3_ Electrolyte

**DOI:** 10.1038/srep28839

**Published:** 2016-06-30

**Authors:** S. Bulja, R. Kopf, A. Tate, T. Hu

**Affiliations:** 1 Bell Labs Ireland, Blanchardstown Industrial Park, Dublin 15, Ireland; 2 Bell Labs, 600 Mountain Ave., Murray Hill, NJ 07974, USA

## Abstract

A great deal of attention has been recently focused on Electrochromic (EC) materials and EC based devices, promoting mainly applications related to display technology. In this case, EC based displays are usually actuated by the application of low dc bias voltages, changing their appearance from transparent to opaque. A variety of studies related to the optical characteristics of EC materials have been reported, however, no serious studies so far have been reported on the possible high frequency tunability of the dielectric characteristics of these materials, with the exception of the work by Rose, which presented the operation of a microwave shutter based on conductive polymers operating in the X-band. Here we report tuneable high frequency dielectric characteristics of an Electrochromic (EC) cell with a complimentary structure of Conductor/WO_3_/LiNbO_3_/NiO/Conductor in the frequency range from 1 GHz to 20 GHz. The EC cell was prepared using standard semiconductor processing technology, such as lithography, etch and deposition techniques. Our measured results indicate that tunability of high frequency dielectric characteristics as a function of dc bias voltage is achieved, and that a possibility exists for this tunability to be tailored.

The property of a change, evocation or bleaching of colour influenced by either an electron-transfer (redox) process or by a sufficient electrochemical potential, referred to as electrochromism, is exhibited by several organic and inorganic materials^1^,^2^,^3^,^4^,^5^,^6^,^7^,^8^,^9^,^10^,^11^,^12^,^13^,^14^. The physical structure of devices that exploit this phenomenon is, in general, multilayered, with each layer deposited separately, Fig. 1. Glass is normally used as the substrate on which the rest of the layers are deposited, as it provides structural stability and is permeable to light; however, other structurally stable substrates can also be used. The optically transparent conductors usually come in the form of Indium Tin Oxide (ITO) and need to be good electrical conductors. The EC film for inorganic based EC materials at the cathode end is usually tungsten oxide (WO_3_), however, a variety of other transition metal oxides can be employed (TiO_2_, MoO_3_, Ta_2_O_5_, Nb_2_O_5_)^12^,^15^,^16^,^17^,^18^ depending of the desired colour of the “ON” or actuated state. It is this layer that contributes to colour modulation based on ion and electron injection and, hence, it needs to be a good electrical conductor. The ion conductor or electrolyte layer serves as a tank of available ions to be injected into the EC film(s). The requirement imposed on this layer is that it needs to display different ion and electron conductivities, typically σ_I_ > 10^−7^ S/cm for ions and σ_e_ < 10^−10^ S/cm for electrons^5^,^12^. The ion storage layer, if present, should display complimentary electrochromic characteristics to the cathodic EC film. Typical transition metal oxides used for this layer are NiO, Cr_2_O_3_, MnO_2_, FeO_2_, CoO_2_, RhO_2_, and IrO_2_. The work presented here aims to explore the possibility of dc bias modulation of the high frequency (1–20 GHz) dielectric characteristics of a traditional solid state EC cell with WO_3_ and NiO as chromic layers and LiNbO_3_ as a solid electrolyte layer. For this purpose, a novel device developed for the high frequency measurements consists of an EC cell, which, in this case, is a finite length microstrip line directly exposed to the composite structure of Conductor/WO_3_/LiNbO_3_/NiO/Conductor, and two broadband coplanar waveguide to microstrip transitions at the two ends of the EC cell. The preparation of the EC cell and the deposition of different layers is appropriately discussed. Upon confirming our initial assumption that dc bias voltage modulation of the high frequency dielectric characteristics takes place alongside optical modulation of the EC cell, we proceed to systematically extract the dielectric characteristics of the EC cell under test. Each step of the extraction procedure is appropriately described. Finally, we discuss the paper in context of the findings.

## Test Device

Essential physics of colouring of WO_3_ and NiO can be represented by the following two, simplified redox equations^11^,^12^,^19^


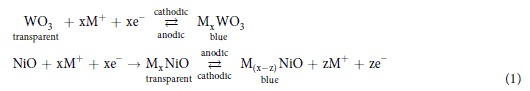


where z ≤ x. Here M stands for the ion of an element of the first group of the periodic system, typically present in the chemical composition of the electrolyte layer. In the present case, M stands for the lithium (Li) ion. From (1) one can see that cathodic darkening of WO_3_ occurs upon ion and electron intercalation, however in the case of NiO, anodic darkening is suggested to be a two stage process^19^. In particular, the intercalated Li^+^ ions “activate” the host structure of NiO, creating Li_x_NiO in the first stage of the process. In the second stage of the process a reversible EC switching occurs between two different phases of the Li-Ni oxide, (1). Effectively, the colouring of NiO occurs upon de-intercalation of ions and electrons from the “actuated” host structure. As mentioned earlier, anodic and cathodic colouring processes in a single EC cell need to be complimentary for improved optical efficiencies, i.e. optical transmittance needs be very high in the bleached state (OFF) and very low in the coloured state (ON).

As evident from the above, optical colouring is induced in EC devices by dc bias voltage, however, the fact that electric charges (ions and electrons) are displaced from the electrolyte layer and subsequently redistributed on the surfaces of the chromic layers intuitively infers that the modulation of the dielectric constant of the EC cell takes place. Due to the causal nature of all dielectric materials, any changes in the dielectric constant need to be followed by a corresponding change in its corresponding loss tangent.

**Figure 2:**
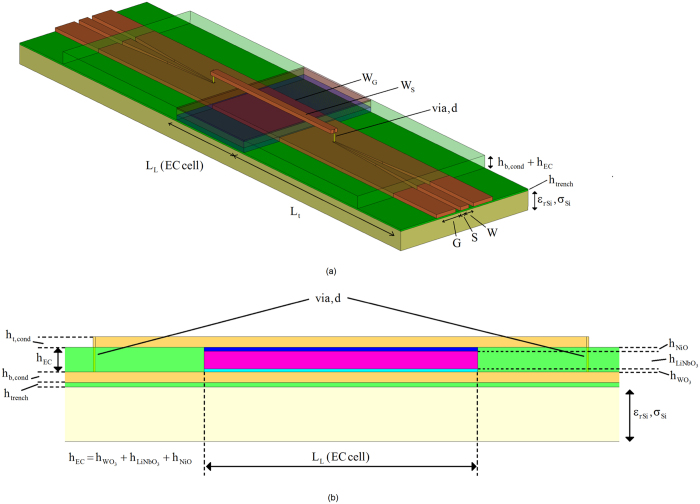
Structure of device (not to scale) for high frequency measurements of dielectric characteristics of EC cell, (a) perspective view and (b) side view of EC cell.

In order to verify that the modulation of high dielectric characteristics of the EC cell takes place, a novel device for broadband measurement of the dielectric properties of the EC cell has been developed; its structure is shown in Fig. 2 20. The EC cell is sandwiched between the ground plane and the microstrip line. At its each end, the microstrip line exposed to the EC material is connected, through a via, to a coplanar waveguide (CPW). The connection formed in this way is, effectively, a broadband transition from microstrip to CPW and its role in the present device is twofold. First, the transitions allow the application of the dc bias voltage, necessary for the actuation of the EC cell and, second, they facilitate the measurements of scattering parameters using a CPW probe station at different dc bias voltages. The transitions are designed to have low reflections.

The device was fabricated using standard processing techniques that are fully compatible with semiconductor device manufacturing. The bottom substrate of the structure of the device shown in Fig. 2 (not to scale), with height h_bs_ = 600 μm is n-doped Si wafer with a surface resistivity of 10 Ω.cm and dielectric constant of ε_rSiO2_ = 11.9. On top of this layer, a thin dielectric layer of SiO_2_ with a height of h_trench_ = 300 nm is deposited so as to separate the conductive Si layer from the deposited gold ground plane. The thickness of the ground plane is h_b,cond_ = 520 nm and it contains the patterned CPW. The CPW is designed to have a 50 Ω characteristic impedance and its geometric parameters are G = 180 μm, S = 44 μm and W = 80 μm, while the length of the broadband transition is L_t_ = 2200 μm. The centre conductor of the CPW is connected, through a via with a diameter of d = 3 μm, to a microstrip line with a width of W_s_ = 4 μm and a length of L_L_ = 5 mm. The top microstrip line is gold, deposited on a SiO_2_ layer with a thickness of h_EC_ = 1μm, which renders its characteristic impedance to be 50 Ω. In this way, the connection between the CPW and the microstrip line is always impedance matched, i.e. the connections exhibits low signal reflections. The thickness of the microstrip line is h_t,cond_ = 2.58 μm. The SiO_2_ layers were deposited using Plasma-Enhanced Chemical-Vapor-Deposition in a Plasma-Therm Shuttle-lock system. They were then lithographically patterned using positive resist on a Suss contact aligner and were subsequently etched using Inductively- Coupled Reactive-Ion Plasma Etching in an SLR770 Plasma-Therm etcher to expose the contact pads and open the vias.

The dimensions of the constituent chromic layers of the EC cell, namely WO_3_ and NiO layers, were chosen in accordance with the relevant studies^11^,^12^,^19^, shown to exhibit the electro-chromic effect. In this study, the respective thicknesses of these layers are h_WO3_ = 150 nm and h_NiO_ = 120 nm. The Au, WO_3_, NiO and LiNbO_3_ layers were also patterned using positive resist on a Suss contact aligner. The resist was then image-reversed to obtain a re-entrant profile for good lift-off. These layers were deposited using e-beam evaporation in an Airco-Temescal evaporator. The evaporator is equipped with an external O_2_ source, which is used during deposition of the WO_3_, NiO and LiNbO_3_ layers to maintain proper stoichiometry. All of the individual layers were characterized using Stylus profilometry on a KLA Tencore P-11 profilometer to determine the thickness. In addition, a Rudolph Auto ELII ellipseometer, and NanoSpec 6100 Interferometer were used to determine the refractive index and the thickness of the individual WO_3_, NiO and LiNbO_3_ layers prior to actual device fabrication. The refractive index obtained was in agreement with published values. The thickness of the electrolyte, LiNbO_3_ layer is chosen so that the overall thickness of the EC cell, h_EC_ = h_WO3_ + h_LiNbO3_ + h_NiO_ is about 1 um, which was necessary to obtain realistic characteristic impedances of the CPW and microstrip sections of about 50 Ω.

## Results

It can be inferred from Fig. 2 that the determination of the dielectric characteristics of the EC cell depends on the knowledge of the scattering (S) or impedance (Z) parameters of the CPW to microstrip transition. The length of this transition is L_t_ = 2200 μm and it extends from the input CPW plane to the microstrip line. Direct measurement of the scattering parameters of this transition is not possible due its split dielectric level and different structures at its input and output, i.e. at one of its inputs the transition has the CPW input, while at its other end the input is in the form of a strip line. However, under the realistic assumption of a single mode propagation regime, the parameters of the transition can be obtained by using the two-tier thru-line (TL)^21^ technique, which relies on the measurements of two passive structures, i.e. the structures which do not contain the EC cell. The first passive structure consists of two transitions of Fig. 2 connected back-to-back, termed thru (T), and two transitions of Fig. 2 connected through a length of a line, termed line (L) exposed to the SiO_2_ layer. The length of the line standard (microstrip line) is chosen to be L = 5 mm.

**Figure 3:**
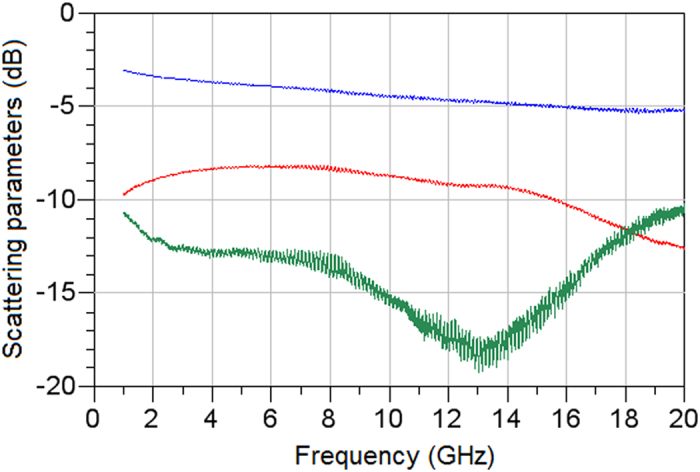
Extracted scattering parameters of CPW to microstrip transition of Fig. 2–transmission coefficient (blue), input (from CPW side) reflection coefficient (red) and output (from microstrip side) reflection coefficient (green).

The scattering parameters of the thru (T) and line (L) standards needed for the extraction of the scattering parameters of the CPW to microstrip transitions, were obtained using a probe station and a vector network analyzer calibrated with a standard short-open-load-thru (SOLT) method. The extracted scattering parameters of the transition are shown in Fig. 3.

The transition is designed to provide good output impedance match from 1 GHz to 20 GHz, since this will allow majority of RF power at the inputs of the CPWs to reach the EC cell under test. This is evidenced in the low reflection coefficient at the output port, Fig. 3. The reflection coefficient, in general, is proportional to a weighted difference between the input impedance of the device and the characteristic impedance of the transmission medium, i.e.:


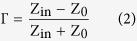


Here, Z_in_ and *Z*_0_ stand for the input impedance and for the characteristic impedance of the transmission medium, respectively. For low reflections (2) states that the difference between impedances Z_in_ and Z_0_ needs to be small. In other words, Z_in_ and Z_0_ are well matched to each other. In most applications, Z_0_ can be considered constant and dependent only on the geometric and the dielectric parameters of the transmission medium, whereas Z_in_ is usually a strong function of not only the geometric and dielectric parameters of the transmission medium, but of frequency too. Further, in most applications, a reflection coefficient below −10 dB is considered to render a good impedance match.

With reference to Fig. 3, it can be appreciated that an input impedance match of better than −8 dB was achieved, while an impedance match better than −10 dB was achieved at the output port. A resonant dip evident in the output reflection coefficient at 12.5 GHz can be attributed to the frequency dependence of Z_in_, which, at a certain frequency is a very close match to Z_0_. This effect is common in applications involving the design of impedance transformers and matching circuits.

Once the scattering parameters of the transitions are obtained, the unknown, effective parameters of the EC cell are obtained from the following matrix expression:


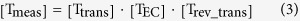


where, [T_meas_] represents the overall measured transmission matrix of the device of Fig. 2, obtained by a conversion of the measured scattering parameters to the transmission parameters. Similarly, [T_trans_] and [T_rev_trans_] represent the extracted transmission matrices of the transition of Fig. 2 for the forward and reverse direction, respectively. The extracted transmission matrix, [T_EC_], contains information of the unknown complex propagation constant of the EC cell, γ_meas_ = α_meas_ + jβ_meas_, however, it also contains the effect of the impedance transition from the microstrip line on the SiO_2_ layer to the unknown dielectric characteristic of the EC material, Fig. 2. The effect of this transition is best evidenced in the fact that the transmission coefficients 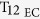 and 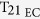 of the extracted unknown transmission matrix [T_EC_] are not equal to zero, but have a finite value. In order to extract the dielectric characteristics of the unknown dielectric material accurately, the effect of this transition needs to be taken into account.

For a symmetrical microstrip line the following holds


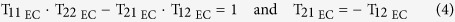


which leads to the following expression for the unknown complex propagation constant obtained using the measured transmission matrix, [T_EC_]


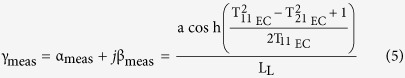


The expression given by (5) represents the extracted complex propagation constant of the EC cell when the effect of the transition from the SiO_2_ layer to the unknown dielectric characteristic of the EC material is taken into account. Effectively, in this way, the microstrip line exposed to the EC material becomes reflection-less and is uniquely characterized by the propagation constant, γ_meas_.

The extracted complex propagation constant, γ_meas_ of (5) represents the effective attenuation and propagation constant, which includes the effect of the electrically thin (smaller than skin depth) bottom and top conductors of the EC cell, Fig. 2. Of particular detrimental influence to the extraction of the dielectric parameters of the EC cell is the bottom gold ground plane, whose thickness of 520 nm is about five times lower than its skin depth at 1 GHz, which makes the ground plane semi-transparent to EM waves at this frequency. In order to obtain precise values of the high frequency dielectric characteristics of the EC cell, exact, measured dimensions of the cell thickness are needed. They are obtained by the use of Scanning Electron Microscopy (SEM), and used in conjunction with a Finite Integration Technique (FIT) available in a 3D numerical simulator^22^ to obtain the match between the computed and measured propagation constant, from which the unknown high frequency dielectric characteristics of the EC cell are obtained.

**Figure 4:**
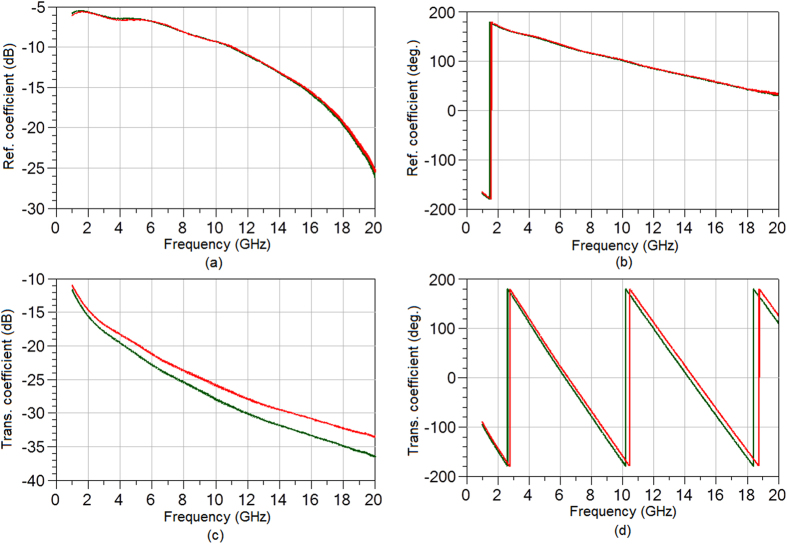
Measured scattering parameters of measurement device of Fig. 2 for 0 V (red) and 6 V (green). (**a**) Magnitude of reflection coefficient, (**b**) Phase of reflection coefficient, (**c**) Magnitude of transmission coefficient and (**d**) Phase of transmission coefficient.

**Figure 5:**
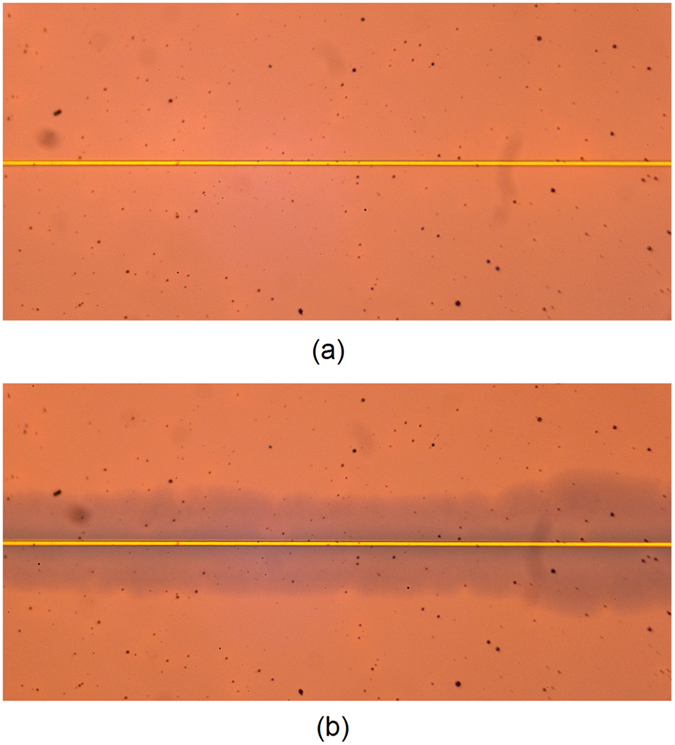
Top view photographs of EC cell for (a) bleached state, 0 V and (b) coloured state, 6 V. Width of golden microstrip line is 4 um.

A dc signal is then applied to the EC cell through a wideband bias tee, connected to one of the probes of the probe station. The scattering parameters of the overall measurement device of Fig. 2 were measured for two bias states, 0 V and 6 V, Fig. 4. Photographs of the EC cell for the bleached (0 V) and coloured state (6 V) are shown in Fig. 5. Due to the fact that the top conductor of the present EC cell is gold with a thickness of 2.58 μm, it is not transparent to visible light, actuation of the EC cell upon application of dc voltage is visually verified with reference to the colour change on the sides of the microstrip line. Upon actuation, the EC cell changes colour from transparent (colouring in Fig. 5a comes from a 520 nm thin gold ground plane deposited on a Si wafer) to blue, Fig. 5b. The colouring of the actuated EC cell in Fig. 5b is strongest in the proximity of the microstrip line and gradually fades away with distance. The EC cell will remain in the actuated state for an extended period of time, determined by choice of electrolyte and chromic layers. This time is usually measured in hours and in some instances days and weeks.

**Figure 6:**
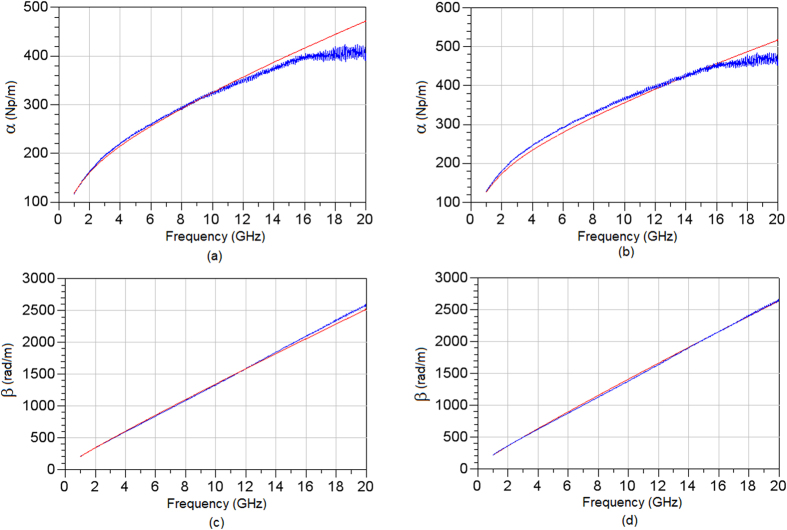
Comparison of extracted (red) and measured (blue) attenuation constants at 0 V (a) and 6 V (b) and propagation constants at 0 V (c) and 6 V (d).

From the measurements of the scattering parameters of Fig. 4, the unknown complex propagation constant *γ*_*meas*_ was extracted using (5) and numerically matched to the computed value of the complex propagation constant. Figure 6 depicts the measured and computed components of the complex propagation constants. As evident, a good match between the measured and computed propagation constants is achieved. The highest error is recorded for the computed attenuation constant, and in the frequency region between 1 GHz–20 GHz, it stands at 10%. It is believed that this error is due to manufacturing imperfections, since the top conductor of the measurement device and hence the EC cell was deposited by e-beam evaporation over the total step height of the active region, ~1 μm. Therefore, the top conductor was somewhat thicker in these areas, which may have contributed to the increased error at high frequencies due to the increased fringing fields in these regions.

**Figure 7:**
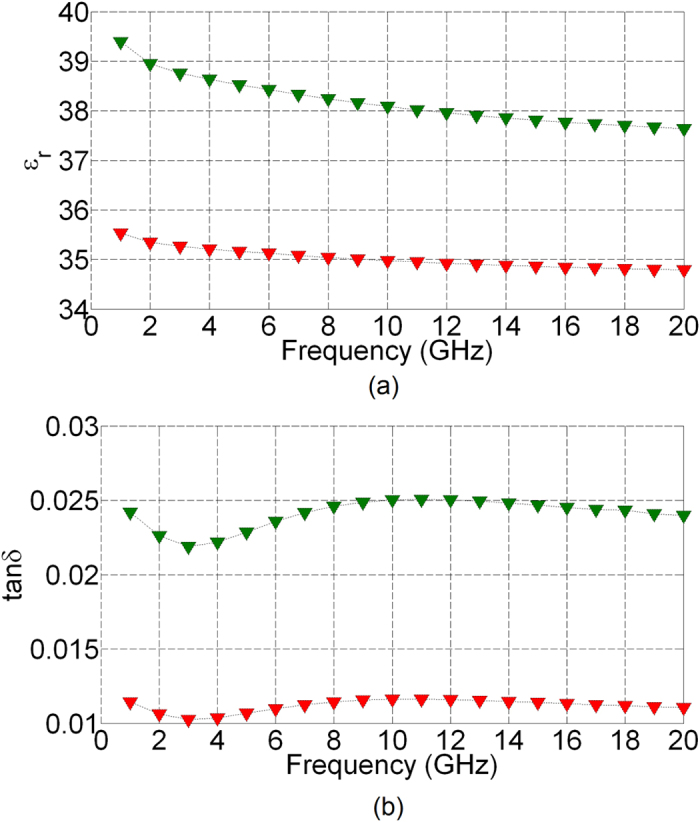
Extracted (a) relative dielectric permittivity of EC cell of Fig. 2 at 0 V (red) and 6 V (green) and (b) loss tangent of EC cell of Fig. 2 at 0 V (red) and 6 V (green).

The dielectric characteristics of the EC cell, ε_r_ and tan(δ), can now be inferred from the knowledge of the extracted propagation constant. Figure 7 presents the extracted dielectric characteristics of the WO_3_/LiNbO_3_/NiO/EC cell for 0 V and 6 V in the frequency range from 1 GHz to 20 GHz. It can be appreciated from these figures that the modulation of high frequency dielectric properties with dc bias voltage takes place. In particular, the dielectric permittivity shows a birefringence varying from 10.8% at 1 GHz to 8.2% at 20 GHz, whereas the loss tangent birefringence is over 200% across the indicated frequency range. It would be pertinent to compare the obtained dielectric characteristics of the EC cell to a corresponding, low dc bias voltage, bulk tuneable technology, such as nematic liquid crystals (LCs). The absolute values of the loss tangent are lower than those reported for the case of LCs^23^,^24^,^25^,^26^, which reported values of loss tangents in the perpendicular state (OFF state) in the range of 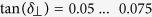 and in the parallel state (ON state) 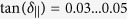 at 30 GHz. Similarly, the absolute values of the dielectric constant of the obtained EC cell are approximately an order of magnitude higher than those reported for the case of LCs^23^,^24^,^25^,^26^, which lie in the range of ε_r⊥_ = 2.75 ... 2.82 and 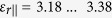 at 30 GHz for the perpendicular and parallel state, respectively. However, in the case of EC materials, it is believed that the absolute values of the dielectric characteristics can be tailored by an appropriate choice of the chromic layers and the electrolyte, which would render them suitable for a variety of tuneable micro-wave, millimetre-wave and opto-high frequency applications. Furthermore, the fact that EC materials exhibit open circuit memory is another added advantage over LCs. Examples of devices which would be enabled by appropriate dielectric tailoring are phase shifters, attenuators, tuneable filters and antennas, to name but a few. This is the subject of our ongoing research, which, coupled with the possibility of optical actuation of the EC cell is particularly appealing.

Another interesting aspect which might be of interest of pursuing lies with the manipulation of orbital occupancy^27^ of thin, transition metal oxide films. The application of a strong electric field (greater than 150 MV/m) induced chemical changes in 20 nm thin (La, Sr)MnO_3-δ_ films^27^ by driving native ions and electrons in and out of the film. In view of this, it may be pertinent to examine if this effect takes place in thin WO_3_ and NiO EC films, which could be, at a later stage combined, with the EC effect. Of particular importance at this stage lies with the fact that the manipulation of orbital occupancy^27^ of thin films occurs at high electric fields, whereas the EC effect takes place at much lower electric fields (3–7 MV/m). This might require careful experiment design.

## Discussion

In this paper, we have demonstrated the existence of tuneable high frequency dielectric characteristics of inorganic EC materials. The dielectric properties of a sample EC cell based on the complimentary structure of Conductor/WO_3_/LiNbO_3_/NiO/Conductor are extracted in a systematic way in the frequency range from 1 GHz to 20 GHz. For this purpose, a unique microwave, CPW to microstrip transitions are developed enabling dc biasing of the test EC cell, and also easing the process of the extraction of the high frequency dielectric characteristics.

Upon visually confirming the actuation of the EC cell by dc bias voltage, we proceed with the extraction of the effective propagation constant of the fabricated EC cell. Here, we discuss a number of challenges needed to be addressed prior to the extraction of the dielectric characteristics of the EC cell. The most detrimental obstacle for successful characterization of the dielectric properties of the EC cell lies with the electrically thin ground plane, which for lower frequencies (below 1 GHz) is semi-transparent to Electro-Magnetic (EM) waves. This effect can only be taken accurately into account, by having the exact knowledge of its thickness and its electrical conductivity.

Finally, we present a short discussion on the obtained dielectric characteristics of the present EC cell and the way it compares with a more mature, low dc bias voltage, bulk tuneable technology, such as LC media. Our work indicates that the present EC cell, which is based on LiNbO_3_ as the solid electrolyte layer, has the extracted “ON” and “OFF” dielectric permittivities that are about an order of magnitude higher than those of LCs, whereas the absolute values of the loss tangents are lower than their LC counterparts. Further, our results indicate that that the high frequency tunability of the dielectric permittivity is about 10%, which is well in-line with the reported dielectric tunability of LC materials. There exists a possibility that the absolute values of the dielectric permittivities and their tuning ranges can be tailored by an appropriate choice of the chromic layers and the electrolyte. Nevertheless, an important finding that arises from this research work lies with the fact that dc bias induced optical modulation of EC materials not only induces changes in the optical appearance of the EC cell, but also its high frequency dielectric characteristics. This offers possibilities for the creation of a family of new devices, capable of exploiting this effect.

## Additional Information

**How to cite this article**: Bulja, S. *et al*. High Frequency Dielectric Characteristics of Electrochromic, WO_3_ and NiO Films with LiNbO_3_ Electrolyte. *Sci. Rep.* **6**, 28839; doi: 10.1038/srep28839 (2016).

## Figures and Tables

**Figure 1:**
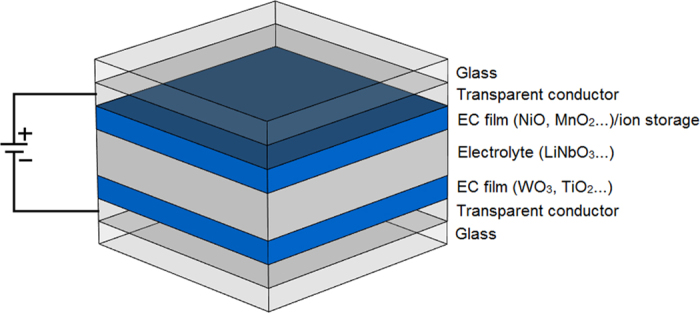
Typical structure of an electrochromic device.
